# The Impact of COPD in Trends of Urinary Tract Infection Hospitalizations in Spain, 2001–2018: A Population-Based Study Using Administrative Data

**DOI:** 10.3390/jcm9123979

**Published:** 2020-12-09

**Authors:** Javier de Miguel-Diez, Romana Albaladejo-Vicente, Domingo Palacios-Ceña, David Carabantes-Alarcon, José Javier Zamorano-Leon, Marta Lopez-Herranz, Ana Lopez-de-Andres

**Affiliations:** 1 Respiratory Department, Hospital General Universitario Gregorio Marañón, 28040 Madrid, Spain; javier.miguel@salud.madrid.org; 2 Instituto de Investigación Sanitaria Gregorio Marañón (IiSGM), Facultad de Medicina, Universidad Complutense de Madrid, CIBER de Enfermedades Respiratorias (CIBERES), 28040 Madrid, Spain; 3 Department of Public Health & Maternal and Child Health, Faculty of Medicine, Universidad Complutense de Madrid, 28040 Madrid, Spain; dcaraban@ucm.es (D.C.-A.); josejzam@ucm.es (J.J.Z.-L.); anailo04@ucm.es (A.L.-d.-A.); 4 Department of Physical Therapy, Occupational Therapy, Rehabilitation and Physical Medicine, Universidad Rey Juan Carlos, Alcorcon, 28922 Madrid, Spain; domingo.palacios@urjc.es; 5 Faculty of Nursing, Physiotherapy and Podology, Universidad Complutense de Madrid, 28040 Madrid, Spain; martal11@ucm.es

**Keywords:** urinary tract infection, COPD, men, women, incidence, in-hospital mortality, Spain

## Abstract

(1) Background: To examine trends in incidence and outcomes of urinary tract infections (UTIs) among men and women with or without chronic obstructive pulmonary disease (COPD), and to identify the predictors for in-hospital mortality (IHM). (2) Methods: We included patients (aged ≥40 years) who were hospitalized with UTIs between 2001 and 2018. Data were collected from the Spanish National Hospital Discharge Database. (3) Results: We identified 748,458 UTI hospitalizations, 6.53% with COPD. The UTIs incidence increased over time. It was 1.55 times higher among men COPD patients than among non-COPD men (incidence rate ratio (IRR) 1.55; 95% CI 1.53–1.56). The opposite happened in women with COPD compared to non-COPD women (IRR 0.30; 95% CI 0.28–0.32). IHM was higher in men with COPD than non-COPD men (5.58% vs. 4.47%; *p* < 0.001) and the same happened in women (5.62% vs. 4.92%; *p* < 0.001). The risk of dying increased with age and comorbidity, but the urinary catheter was a protective factor among men (OR 0.75; 95% CI 0.64–0.89). Multivariable analysis showed a significant reduction in the IHM over time for men and women with COPD. Suffering from COPD only increased the risk of IHM among men (OR 1.07; 95% CI 1.01–1.13). (4) Conclusions: The incidence of UTIs increased over time. Suffering COPD increased the risk of IHM among men, but not among women.

## 1. Introduction

Chronic obstructive pulmonary disease (COPD) is a major cause of morbidity and mortality, with high economic and social cost [1]. It is the fourth most common cause of death worldwide, and it is expected to be the third by the end of 2020 [2]. In addition, the global burden of this disease is expected to increase in the coming years, due to increasing tobacco use in women and aging of the world population [1].

COPD is defined by a single physiologic parameter, spirometry. However, it exhibits a significant clinical heterogeneity [3]. Recent investigations have also reported the necessity to view COPD in the context of common comorbidities [4]. 

The presence of comorbidities between patients with COPD is high [5]. In this way, adults with COPD generally have a higher burden of comorbid conditions than those without COPD. For example, a case-control study reported that COPD patients had, on average, 3.7 comorbidities compared to 1.8 in individuals without COPD [6]. Some of these comorbidities are related to aging, and others share the underlying mechanisms (e.g., systemic inflammation) or the risk factors (e.g., smoking exposure) [7]. Furthermore, comorbidities contribute to a decrease in health status and functional performance, as well as an increase in healthcare resources utilization and mortality [8].

The most prevalent comorbidities in people with COPD are cardiovascular, metabolic, musculoskeletal and psychological [9]. However, both everyday practice and literature searches provide evidence of other, less-recognized diseases, which can be associated with COPD. Among the urogenital comorbidities, chronic kidney disease and benign prostatic hyperplasia have been reported as the main conditions associated with this chronic pulmonary disease [10]. On the other hand, urinary tract infections (UTIs) are a comorbidity that is insufficiently recognized [11], with symptoms being neglected as being difficult to differentiate from symptoms of complications or side effects of therapy [12].

UTIs are one of the most frequent bacterial infections in the community and in hospitals, and they are associated with high mortality, morbidity, length of hospital stay (LOHS) and health costs [13]. 

Therefore, their prevention and management in COPD-hospitalized patients could provide benefits in reducing the global load of the disease, especially since international recommendations on management of this disease do not systematically include the evaluation of comorbid conditions in its diagnostic approach or in the treatment decisions, focusing on isolated lung impairment rather than multimorbidity [14].

In this study, we used national hospital discharge administrative data to examine trends in incidence and outcomes of UTIs among men and women with or without COPD in Spain from 2001 to 2018. In particular, we analyzed patient comorbidities, procedures, UTIs’ pathogens and in-hospital outcomes, such as in-hospital mortality (IHM) and LOHS. Finally, we identified the predictors for IHM after hospitalization with UTIs among men and women with COPD.

## 2. Materials and Methods

### 2.1. Study Design and Data Collection

We conducted a retrospective observational study using the Spanish National Hospital Discharge Database (SNHDD). We included all hospital admissions between 1 January 2001 and 31 December 2018. The SNHDD is an administrative database that collects de-identified demographic, clinical and resource utilization data of all public and private Spanish hospitals. Every year, over 98% of Spanish hospitals send their data to the Ministry of Health, which freely provides the requested databases to the investigators [15].

The principal and secondary diagnosis and the therapeutic and diagnosis procedures conducted during the hospital admission are codified using the International Classification of Diseases (ICD). From 2001 to 2015, the SNHDD used the 9th Revision Clinical Modification (ICD-9-CM) that was replaced by the 10th Revision (ICD-10) from 2016 onwards. Details of the SNHDD database can be found elsewhere [15].

We selected all admissions for patients aged 40 years or over with a primary diagnosis of UTI based on the definition of the Agency for Healthcare Research and Quality (AHRQ) Prevention Quality Indicator 12 for Urinary Tract Infections [16]. We grouped admissions by COPD status as follows: COPD (ICD-9-CM codes: 490, 491, 491.0, 491.1, 491.2x, 491.8, 491.9, 492, 492.0, 492.8 and 496; ICD-10: J44.0, J44.1, and J44.9) in any diagnostic position or non-COPD for those without any of these codes. According to the SNHDD, the primary diagnosis is the main condition that caused the hospitalization of the patient, and therefore must always be present on admission [15].

### 2.2. Study Variables

The study variables are the incidence of UTIs per 100,000 inhabitants and hospital outcome variables such as LOHS and IHM. IHM is defined by the proportion of patients who died during admission for each year of study.

For each hospital admission, we analyzed the sex and age of the patients as demographic variables. To describe the clinical profile, we used the conditions included in the Charlson Comorbidity Index (CCI), using the algorisms for administrative databases using ICD 9 and ICD 10 codes described by Quan et al. [17]. The presence of isolated microorganisms assessed using the ICD codes are shown in Supplementary Table S1. We specifically identified patients with codes for *Enterococcus, Staphylococcus aureus, Klebsiella pneumoniae, Escherichia coli, Proteus* and *Pseudomonas aeruginosa*, in any diagnosis field. According to the SNHDD, only pathogens that have been laboratory confirmed can be included in the database [15].

Finally, the variable “Urinary catheter” was created using the procedure codes described in Table S1.

### 2.3. Statistical Methods

All statistical analysis is presented separately for men and women. The time period from 2001 to 2018 was analyzed in six three-year periods. 

The incidence rates of admission for UTIs among COPD patients and non-COPD patients per 100,000 inhabitants were estimated using the methods described in previous studies [18]. Poisson regression models adjusted by age–sex were used to assess the time trends for study groups providing incidence rate ratios (IRR) with 95% confidence interval as the measure of association.

Sample characteristics were described using proportions for categorical variables and mean and standard deviation (SD) or median and interquartile range (IQR) for continuous variables. To compare proportions, we used Chi-square test, Student *t*-test for means and Wilcoxon–Mann–Whitney test for medians.

Time trend for study variables was assessed using bivariate logistic regression (proportions), ANOVA (means) or Kruskal–Wallis test (medians), as appropriate.

A multivariable logistic regression model was constructed to identify predictors of IHM among COPD patients with UTIs providing odds ratios (ORs) with 95% CI. Finally, using the entire database, we analyzed the effect of COPD on the IHM. 

Stata version 14 (Stata, College Station, TX, USA) was used for data analysis.

### 2.4. Ethical Aspects

According to the Spanish legislation, as we used the SNHDD, which is a de-identified retrospective public access database that is provided freely to all investigators by the Spanish Ministry of Health, it was not necessary to obtain approval by an ethics committee or informed consent by the patients. 

## 3. Results

We identified a total of 748,458 hospitalizations of patients aged ≥40 years old with a primary diagnosis of UTIs (6.53% with COPD) in Spain between 2001 and 2018. In COPD patients who had an admission for UTIs, there was a significant male predominance (79.6%), whereas in non-COPD patients, there was a significant female predominance (58.6%).

### 3.1. Incidence of UTIs According to COPD

The detailed incidence rates per 100,000 inhabitants according to COPD status, age groups and sex in Spain from 2001 to 2018 are shown in Table S2.

The incidence of UTIs coding increased in both men and women, with and without COPD, significantly from 2001 to 2018 (all *p* < 0.001). Equivalent trends in the figures for men and women without COPD were found ((Supplementary Table S2). In COPD patients, the incidence was significantly higher in men than in women for all years analyzed; however, in non-COPD patients, the incidence was higher in women. Over the entire time period, the incidence in men with COPD was 237.6, and in women with COPD it was 58.01 (*p* < 0.001). Among COPD patients, overall and in men and women separately, the incidence increased with age in all time periods. For the entire time period, the incidence was highest among men and women aged ≥85 years. 

**Figure 1:**
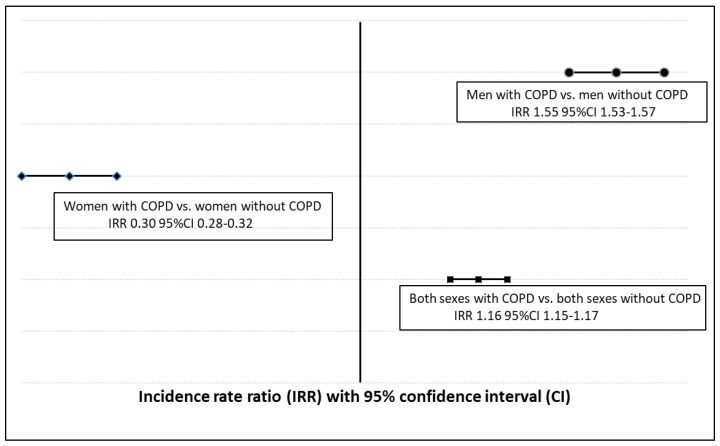
Forrest plot showing adjusted Incidence Rate Ratios for Urinary Tract Infections comparing men and women with and without COPD in Spain from 2001 to 2018. Incidence Rate Ratios obtained with Poisson regression adjusted by age and sex as appropriate.

The results of the Poisson regression models are shown in Figure 1. The adjusted incidence of UTIs in men was 1.55 times higher among patients with COPD than among those without COPD (IRR 1.55; 95% CI 1.53–1.56). However, these models show opposite figures for women with COPD (IRR 0.30; 95% CI 0.28–0.32) when compared to non-COPD women. When both sexes are merged, the IRR is 1.16 (95% CI 1.15–1.17).

Characteristics of UTIs among men and according to the presence of COPD. The age distribution, comorbidities, procedures, in-hospital outcomes and isolated pathogens among men hospitalized with a principal diagnosis of urinary tract infection in Spain from 2001 to 2018, according to COPD status, are shown in Table 1.

Overall, men with COPD were older (78.59; SD = 9.11 years) than patients without COPD (73.03; SD = 13.12 years; *p* < 0.001) and had more coexisting medical conditions (mean CCI index: 1.2 ± 0.93 vs. 1.04 ± 0.89; *p* < 0.001) and required urinary catheter more frequently (8.59% vs. 7.45%; *p* < 0.001). 

Regarding hospital outcomes, the overall median LOHS was 6 days in men with and without COPD (*p* = 0.453). For the total time period, crude IHM was higher in men with COPD than non-COPD men (5.58% vs. 4.47%; *p* < 0.001).

Among men, the most frequently isolated pathogens were *Escherichia coli* (COPD: 25.27% and non-COPD: 25.41%; *p* > 0.05), *Pseudomonas aeruginosa* (COPD: 6.04% and non-COPD: 5.58%; *p* < 0.001) and *Enterococcus* (COPD: 5.32% and non-COPD: 4.56%; *p* < 0.001). No difference was found for isolations of *Staphylococcus aureus* (COPD: 2.62% and non-COPD: 2.59%; *p* = 0.723) and *Klebsiella pneumoniae* (COPD: 5.08% and non-COPD: 5.18%; *p* = 0.435).

When we analyzed the time trend from 2001 to 2018 (Table S3), we observed that the age, comorbidity and use of urinary catheter increased significantly over time in both men with and without COPD. However, IHM decreased significantly from 6.47% and 4.92% in 2001/3 for men with and without COPD to 5.34% and 4.19% in 2016/8, respectively (both *p* < 0.001). Regarding isolated pathogens, all become more frequently codified over time among men with UTIs beside the presence of concomitant COPD (Table S4).

### 3.2. Characteristics of UTIs among Women and According to the Presence of COPD

As can be seen in Table 2, overall, COPD women were older (79.89 vs. 75.56 years; *p* < 0.001), had more comorbidity (mean CCI: 1.17 vs. 1.01 *p* < 0.001) and higher frequency insertion of urinary catheter (3.78% vs. 3.38% *p* < 0.001) during UTIs admission than women without COPD. For the total time period, crude IHM was 5.62% for women with COPD and 4.92% for non-COPD women (*p* < 0.001).

The most prevalent pathogens isolated among women were *Escherichia coli* (COPD: 36.51% and non-COPD: 33.97%; *p* < 0.001), *Klebsiella pneumoniae* (COPD: 4.94% and non-COPD: 4.78%; *p* = 0.465) and *Enterococcus* (COPD: 3.38% and non-COPD: 2.9%; *p* = 0.005).

Beside the presence of COPD, women have increased their mean age, their mean CCI and the frequency of urinary catheter over the study period (all *p* < 0.001). Median LOHS and IHM decreased significantly over time in women with COPD, though in women without COPD, the IHM increased over time (Table S3).

Time trends in the prevalence of isolated pathogens among women with COPD showed a significant increment for *Enterococcus*, *Klebsiella pneumoniae* and *Escherichia coli*, with no variation over time for the other three pathogens described. Among non-COPD women, all pathogens’ isolations increased, with the exception of *Staphylococcus aureus*, which decreased.

### 3.3. Variables Associated with IHM among Men and Women with UTIs

The variables associated with IHM after multivariable analysis in patients with COPD are shown in Table 3.

For both sexes, the risk of dying increased with age and with suffering a higher number of conditions included in the CCI.

The insertion of a urinary catheter was a protective factor among men (OR 0.75; 95% CI 0.64–0.89).

Regarding isolated pathogens, *Escherichia coli*, *Klebsiella pneumoniae* and *Proteus* were associated with a lower risk of dying during the hospitalization in both sexes. In men, the presence of *Pseudomonas aeruginosa* was also associated with a lower risk. However, isolation of *Staphylococcus aureus* increased the risk of IHM by 50% among men (OR 1.5; 95% CI 1.2–1.87).

After adjusting for study variables, the result of the multivariable analysis showed a significant reduction in the IHM over time for men (OR 0.91; 95% CI 0.89–0.94) and women (OR 0.91; 95% CI 0.86–0.96) with COPD.

When we analyze the entire database for men, women and both sexes and conduct multivariable logistic regression to identify variables associated with IHM, we obtain the results shown in Table 4. After adjusting for all the study variables, suffering COPD increased the risk of IHM among men admitted to the hospital with an episode of UTIs (OR 1.07; 95% CI 1.01–1.13), but not among women (OR 0.99; 95% CI 0.91–1.08).

## 4. Discussion

Our analysis showed that, among hospitalized patients with UTIs in Spain between 2001 and 2018, 6.53% had COPD. Previous researchers have pointed out that UTI is an insufficiently recognized comorbidity among patients with COPD [11]. However, in a recent study in which 580 patients with COPD were analyzed, it was found that 218 (37%) had comorbid UTIs [11].

In the present investigation, the incidence of UTI admissions increased significantly over the study period in men and women with and without COPD. Age, comorbidity and use of urinary catheter increased significantly over time in both groups of patients, which may have contributed to these results. Zilbelberg et al. also found that, between 2000 and 2009, the frequency of UTI hospitalizations increased by approximately 50% in the US, from 53 to 77 cases per 1000 hospitalizations [19]. Other studies have also provided evidence indicating that hospital admission for UTI is increasing in Europe [20] and the US [21], and thus UTI is becoming an important cause of health service use in older adults.

In non-COPD patients, the incidence of UTI was higher in women, which is consistent with well-established evidence of higher risk for UTIs among females [22,23]. However, in COPD patients, the incidence was significantly higher in men than in women for all years analyzed. In previous studies, it has been described that UTI in men is uncommon, except when abnormalities (such as prostatic enlargement or a urethral stricture) or invasive procedures in the urinary tract are present [24,25,26]. Another factor that can influence the development of UTIs in these patients is the treatment received for COPD. For example, the most frequently reported genitourinary complications of inhaled anticholinergics are urinary difficulty, urinary retention, and urinary tract infection [27]. In addition, it has been demonstrated that older men with COPD newly started on inhaled, long-acting anticholinergic medication (LAA) are at increased risk of UTI [28]. By gender, men newly started on this treatment were 75% more likely to develop a UTI than men newly started on an inhaled corticosteroid. However, no significant association was seen in women. Thus, men considering an inhaled LAA should be informed of this risk and, if they decide to take it, be provided with appropriate monitoring [28]. Furthermore, in a recent study, Savaria et al. [29] found that 15% of men with COPD newly treated with LAA suffered from benign prostatic hyperplasia.

For both men and women with and without COPD incidence of UTIs, admissions increased with age, the highest being among patients aged >85 years. Thus, older patients seem to be disproportionally contributing to the increase in hospitalizations [30]. They often have coexisting conditions, such as diabetes mellitus, that are associated with an increased susceptibility to infection. Urologic coexisting conditions, such as incontinence or urinary retention, facilitate the acquisition of bacteriuria owing to an increased exposure to interventions such as catheterization [31].

As most epidemiologic studies of UTI have identified [32], we found that *E. coli* was the main infection pathogen, both in patients with COPD and in those without this disease. In relation to patients with COPD, Nikolié et al. also found that *E. coli* was responsible for 85.5% of UTIs in patients with grades II and III of this disease [11]. On the other hand, as described by other authors [19], the prevalence of all the pathogens analyzed increased significantly over time in our investigation, except for *S. aureus*, which tended to remain stable during the study period in COPD patients and decreased significantly over time in those without COPD.

Crude IHM was higher in men and women with COPD than in those without COPD, and it decreased significantly over time in all groups except women without COPD. In a previous study, a decrease in in-hospital mortality has also been observed in patients with COPD over time; no differences between men and women having been evaluated [33]. In both sexes, the risk of dying increased with age and with suffering any of the conditions included in the CCI. Older age and CCI have been previously identified as risk factors for mortality in patients with UTI [34]. However, we observed that the insertion of a urinary catheter was a protective factor of IHM among men. In this way, in a multicenter, multinational, retrospective cohort study, Gomila et al. observed that, although catheter-associated UTI was the most frequent source of complicated UTI, typically involving older males with more comorbidities and multidrug-resistant Gram-negative bacteria, after adjusting for confounders, catheter-associated UTI was not independently associated with an increased risk of mortality [35].

In our study, after multivariable analysis, suffering COPD increased the risk of IHM among men admitted to the hospital with an episode of UTI, but not among women. Mitchell et al. also found that women with healthcare-associated UTIs were less likely to die [36]. In any case, we found a significant reduction in IHM in women with COPD, though it increased over time in women without COPD. This finding may be due to the fact that the greater HMI in the non-COPD females is actually related to non-UTI related death, which should be clarified in further studies.

In our investigation, an increased risk of IHM associated with UTI by *Staphylococcus aureus* was found. It has been previously described that, in up to 34% of cases, *Staphylococcus aureus* bacteriuria is associated with bacteremia by this microorganism. These patients frequently have a complicated course with higher hospital mortality [37].

We found a decrease in LOHS over time in male COPD patients. Factors that have been associated with LOHS in patients with COPD include age, disease severity and the presence of comorbidities [38]. In addition to the decrease in LOHS, we also observed a reduction in IHM, even though we found an increase of age, comorbidity and use of urinary catheter, which indicates that the management of these patients has been improving over time. The strengths of our study are the large number of patients evaluated, increasing the generalizability of our findings, and the use of a standardized methodology that has remained stable over time. However, some limitations should be considered in the interpretation of our results. First, hospital admissions for UTI and COPD were identified using ICD codes and could be subject to misclassification. Second, the SNHDD does not collect information on COPD severity using the GOLD stage or other methods. However, it has been previously reported that COPD stage was not associated with hospitalization for UTI [39]. Furthermore, this registry also lacks data regarding disease duration or specific treatments. On the other hand, we did not have available other data that could be of interest, such as microbiological resistance or the antibiotic treatment used. Moreover, during the long study period, it is also possible that some changes in practices for coding have occurred, although we have not identified any extensive changes in these practices. Finally, we cannot observe what happens to patients after discharge and cannot therefore comment confidently regarding longer-term mortality or healthcare cost implications.

## 5. Conclusions

We observed that the incidence of UTI increased over time in men and women with and without COPD. It was higher among men COPD patients than among non-COPD men, contrary to what happened in women. IHM has reduced significantly over time in COPD patients. Suffering COPD increased the risk of IHM among men, but not among women. Knowledge of this epidemiological pattern can help us improve the early recognition and appropriate management of patients with UTI and COPD and reduce the mortality of patients with both diseases.

## Figures and Tables

**Table 1:** Age distribution, comorbidities, procedures, in-hospital outcomes and isolated pathogens among men hospitalized with a principal diagnosis of urinary tract infection in Spain from 2001 to 2018, according to COPD status.

Variables	Men with COPD (38,971)	Men without COPD (289,008)	*p*
Age, mean (SD)	78.59 (9.11)	73.03 (13.12)	<0.001
40–54 years	539 (1.38)	32,082 (11.10)	<0.001
55–69 years	5526 (14.18)	69,485 (24.04)	
70–84 years	22,248 (57.09)	128,401 (44.43)	
≥85 years	10,658 (27.35)	59,040 (20.43)	
CCI mean (SD)	1.2 (0.93)	1.04 (0.89)	<0.001
CCI = 0	10,807 (27.73)	100,079 (34.63)	<0.001
CCI 1–2	14,735 (37.81)	107,827 (37.31)	
CCI > 2	13,429 (34.46)	81,102 (28.06)	
Urinary catheter, *n* (%)	3348 (8.59)	21,526 (7.45)	<0.001
LOHS, median (IQR)	6 (6)	6 (6)	0.453
IHM, *n* (%)	2175 (5.58)	12,929 (4.47)	<0.001
*Enterococcus*, *n* (%)	2075 (5.32)	13,176 (4.56)	<0.001
*Staphylococcus aureus*, *n* (%)	1021 (2.62)	7483 (2.59)	0.723
*Klebsiella pneumoniae*, *n* (%)	1981 (5.08)	14,962 (5.18)	0.435
*Escherichia coli*, *n* (%)	9849 (25.27)	73,429 (25.41)	0.575
*Proteus*, *n* (%)	953 (2.45)	8279 (2.86)	<0.001
*Pseudomonas aeruginosa*, *n* (%)	2355 (6.04)	16,119 (5.58)	<0.001

COPD; Chronic Obstructive Pulmonary Disease. CCI; Charlson Comorbidity Index. LOHS; Length of Hospital Stay. IQR; Inter Quartile Range. IHM; In-Hospital Mortality. *p* values for differences between those with and without COPD. To compare proportions, we used Chi-square test, Student *t*-test for means and Wilcoxon–Mann–Whitney test for medians.

**Table 2:** Age distribution, comorbidities, procedures, in-hospital outcomes and isolated pathogens among men hospitalized with a principal diagnosis of urinary tract infection in Spain, 2001–2018, according to COPD status.

Variables	Women with COPD (9971)	Women without COPD (410,508)	*p*
Age, mean (SD)	79.89 (10.81)	75.56 (14.45)	<0.001
40–54 years	362 (3.63)	51,722 (12.60)	<0.001
55–69 years	1185 (11.88)	61,694 (15.03)	
70–84 years	4503 (45.16)	165,773 (40.38)	
≥85 years	3921 (39.32)	131,319 (31.99)	
CCI mean (SD)	1.17 (0.88)	1.01 (0.86)	<0.001
CCI = 0	2787 (27.95)	145,512 (35.45)	<0.001
CCI 1–2	3864 (38.75)	153,518 (37.4)	
CCI > 2	3320 (33.3)	111,478 (27.16)	
Urinary catheter, *n* (%)	377 (3.78)	13,868 (3.38)	0.028
LOHS, median (IQR)	6 (6)	6 (6)	0.193
IHM, *n* (%)	560 (5.62)	20,212 (4.92)	0.002
*Enterococcus, n* (%)	337 (3.38)	11,919 (2.9)	0.005
*Staphylococcus aureus*, *n* (%)	166 (1.66)	6308 (1.54)	0.344
*Klebsiella pneumoniae*, *n* (%)	493 (4.94)	19,638 (4.78)	0.465
*Escherichia coli, n* (%)	3640 (36.51)	139,441 (33.97)	<0.001
*Proteus, n* (%)	251 (2.52)	11,042 (2.69)	0.293
*Pseudomonas aeruginosa*, *n* (%)	238 (2.39)	8822 (2.15)	0.114

COPD; Chronic Obstructive Pulmonary Disease. CCI; Charlson Comorbidity Index. LOHS; Length of Hospital Stay. IQR; Inter Quartile Range. IHM; In-Hospital Mortality. *p* values for differences between those with and without COPD. To compare proportions, we used Chi-square test, Student *t*-test for means and Wilcoxon–Mann–Whitney test for medians.

**Table 3:** Variables associated with in-hospital mortality in hospital admissions of COPD patients with a principal diagnosis of urinary tract infection according to sex in Spain (2001–2018).

Variables	Men	Women	Both
	OR (95% CI)	OR (95% CI)	OR (95% CI)
Female sex	NA	NA	0.99 (0.9–1.1)
40–54 years	1	1	1
55–69 years	1.28 (0.66–2.47)	3.56 (0.82–15.54)	1.74 (0.96–3.18)
70–84 years	2.5 (1.32–4.74)	10.04 (2.45–41.18)	3.61 (2.02–6.46)
≥85 years	5.33 (2.82–10.09)	18.27 (4.46–74.85)	7.45 (4.17–13.33)
CCI = 0	1	1	1
CCI 1–2	1.69 (1.48–1.92)	1.47 (1.14–1.88)	1.64 (1.46–1.84)
CCI > 2	2.55 (2.25–2.9)	2.1 (1.65–2.68)	2.45 (2.19–2.74)
Urinary catheter	0.75 (0.64–0.89)		0.8 (0.68–0.94)
*Staphylococcus aureus*	1.5 (1.2–1.87)		1.49 (1.21–1.84)
*Klebsiella pneumoniae*	0.79 (0.63–0.98)		0.81 (0.67–0.98)
*Escherichia coli*	0.48 (0.42–0.54)	0.5 (0.4–0.61)	0.48 (0.43–0.53)
*Proteus*	0.7 (0.51–0.95)		0.69 (0.52–0.9)
Year	0.91 (0.89–0.94)	0.91 (0.86–0.96)	0.91 (0.89–0.93)

OR Odds ratio. CI; Confidence interval. NA; Not adequate. CCI; Charlson Comorbidity Index. Only variables with that remained in the final model after multivariable model construction are shown in the table.

**Table 4:** Variables associated with in-hospital mortality in hospital admissions of patients with a principal diagnosis of urinary tract infection according to sex in Spain (2001–2018).

Variables	Men	Women	Both
	OR (95% CI)	OR (95% CI)	OR (95% CI)
Female sex	NA	NA	1.01 (0.99–1.04)
40–54 years	1	1	1
55–69 years	1.95 (1.71–2.23)	2.81 (2.46–3.22)	2.38 (2.17–2.62)
70–84 years	4.8 (4.24–5.42)	7.51 (6.65–8.5)	6.08 (5.57–6.63)
≥85 years	10.24 (9.05–11.58)	15.36 (13.59–17.36)	12.66 (11.61–13.81)
CCI = 0	1	1	1
CCI 1–2	1.68 (1.60–1.76)	1.46 (1.41–1.52)	1.55 (1.51–1.60)
CCI > 2	2.41 (2.3–2.53)	2.08 (2.00–2.16)	2.21 (2.15–2.28)
Urinary catheter	0.71 (0.67–0.76)	1.15 (1.07–1.23)	0.89 (0.84–0.93)
Staphylococcus aureus	1.47 (1.35–1.60)	1.74 (1.59–1.91)	1.59 (1.50–1.70)
Klebsiella pneumoniae	0.73 (0.67–0.80)	0.76 (0.71–0.82)	0.75 (0.71–0.79)
Escherichia coli	0.51 (0.49–0.54)	0.47 (0.45–0.49)	0.49 (0.47–0.50)
Pseudomonas aeruginosa	0.75 (0.70–0.81)	-	0.86 (0.81–0.91)
Proteus	0.76 (0.68–0.84)	0.87 (0.80–0.94)	0.82 (0.77–0.88)
Year	0.92 (0.91–0.93)	0.95 (0.94–0.96)	0.94 (0.93–0.94)
COPD	1.07 (1.01–1.13)	0.99 (0.91–1.08)	1.01 (0.97–1.06)

OR Odds ratio. CI; Confidence interval. NA; Not adequate. CCI; Charlson Comorbidity Index. Only variables with that remained in the final model after multivariable model construction are shown in the table.

## Supplementary Materials

The following are available online at https://www.mdpi.com/2077-0383/9/12/3979/s1, Table S1: ICD 9 MC and ICD 10 MC codes used to identify diagnosis and procedures used in this investigation, Table S2: Time trends in hospital admissions among patients with a principal diagnosis of urinary tract infection per 100,000 inhabitants in Spain from 2001 to 2018, according to COPD status, age groups and sex, Table S3: Time trends in comorbidities, procedures and in-hospital outcomes among patients with a principal diagnosis of urinary tract infection in Spain from 2001 to 2018, according to COPD status and sex, Table S4: Time trends in isolated pathogens codified in hospital admissions among patients with a principal diagnosis of urinary tract infection in Spain from 2001to 2018, according to COPD status and sex.
