# Supplementary material for: The Impact of COPD in Trends of Urinary Tract Infection Hospitalizations in Spain, 2001–2018: A Population-Based Study Using Administrative Data
Source: J Clin Med. 2020 Dec 9;9(12):3979. doi: 10.3390/jcm9123979 (PMC7763854; doi:10.3390/jcm9123979)
Supplement: Supplementary file 1 [file jcm-09-03979-s001.pdf]

Supplementary Table 1. ICD 9 MC and ICD 10 MC codes used to identify diagnosis and procedures used in this investigation.

**DIAGNOSIS/PROCEDURES**	**ICD 9 CM**	**ICD 10 CM**
Enterococcus	041.04	B95.2; A40.2; A41.81
Staphylococcus aureus	041.1; 041.10; 041.11; 041.12; 041.19; 038.1; 038.10; 038.11; 038.12; 038.19	B95.6; B95.61; B95.62; B95.7; B95.8; A41.0; A41.1; A41.2 A41.01; A41.02
Klebsiella pneumoniae	041.3	B96.1
Escherichia coli	**041.4; 041.41; 041.42; 041.43; 041.49; 038.42**	**B96.21; B96.22; B96.23; B96.20 B96.29; A41.51**
Proteus (mirabilis) (morganii)	041.6	B96.4
Pseudomonas aeruginosa	041.7; 038.43	B96.5; A41.52
Urinary catheter	57.94; 57.95	0T9B70Z; 0T9B80Z; 0T2BX0Z

Supplementary Table 2. Time trends in hospital admissions among patients with a principal diagnosis of urinary tract infection per 100,000 inhabitants in Spain from 2001 to 2018, according to COPD status, age groups and sex.

Sex	Age groups	2001-2003 N (Inc/10^5)	2004-2006 N (Inc/10^5)	2007-2009 N (Inc/10^5)	2010-2012 N (Inc/10^5)	2013-2015 N (Inc/10^5)	2016-2018 N (Inc/10^5)	Total N (Inc/10^5)	p-value
**Men with COPD**	40-54 years	71(17.22)	71(10.11)	66(5.42)	81(9.56)	126(35.6)	124(32.13)	539(13.75)	<0.001
	55-69 years	644(86.23)	736(67.73)	769(73.94)	991(96.83)	1105(178.85)	1281(185.27)	5526(106.14)	<0.001
	70-84 years	2372(237.39)	3028(287.46)	3490(304.58)	4080(402.66)	4512(491)	4766(560.32)	22248(371.97)	<0.001
	≥85 years	806(519.49)	1076(579.95)	1555(579.67)	2006(744.64)	2266(1322.48)	2949(1203.13)	10658(823.15)	<0.001
	Total	3893(168.28)	4911(162.21)	5880(160.12)	7158(226.99)	8009(388.39)	9120(419.68)	38971(237.6)	<0.001
**Men without COPD**	40-54 years	4155(32.41)	4528(32.12)	5150(36.04)	5408(35.92)	6244(38.77)	6597(39.55)	32082(36.03)	<0.001
	55-69 years	7599(91.22)	8609(104.5)	10001(110.28)	11630(120.96)	14604(139.07)	17042(153.63)	69485(122.23)	<0.001
	70-84 years	13082(297.73)	16055(315.21)	20112(422.16)	22308(451.36)	26030(477.17)	30814(522.91)	128401(420.41)	<0.001
	≥85 years	4506(886.95)	5468(1019.24)	7840(1201.46)	10282(1363.43)	13148(1310.55)	17796(1810.09)	59040(1330.46)	<0.001
	Total	29342(112.63)	34660(123.95)	43103(149.79)	49628(163.42)	60026(181.53)	72249(208.52)	289008(159.79)	<0.001
**Women with COPD**	40-54 years	32(6.59)	42(4.12)	52(3.17)	67(6.13)	79(21.5)	90(26.6)	362(7.32)	<0.001
	55-69 years	123(18.33)	111(11.02)	153(11.03)	205(19.08)	241(33.4)	352(47.29)	1185(21.14)	<0.001
	70-84 years	533(69.25)	557(55.09)	719(58.02)	835(82.68)	879(141.15)	980(127.24)	4503(83.04)	<0.001
	≥85years	320(217.75)	389(330.65)	577(246.87)	775(299.82)	809(325.68)	1051(496.75)	3921(322.24)	<0.001
	Total	1008(48.63)	1099(34.83)	1501(33.36)	1882(54.78)	2008(102.44)	2473(119.79)	9971(58.01)	<0.001
**Women without COPD**	40-54 years	6034(48.03)	6927(50.02)	7980(57.45)	8705(59.32)	10509(66.22)	11567(69.74)	51722(59.16)	<0.001
	55-69 years	8129(87.71)	8646(90.14)	9048(95.5)	10433(102.14)	12213(105.88)	13225(112.04)	61694(99.69)	<0.001
	70-84 years	17866(269.24)	22028(314.33)	25732(378.65)	29993(416.88)	33171(460.5)	36983(482.43)	165773(390.03)	<0.001
	≥85years	8915(790.55)	11470(1089.09)	17425(1164.05)	23682(1435.15)	29951(1570.41)	39876(1844.18)	131319(1397.4)	<0.001
	Total	40944(138.35)	49071(155.78)	60185(190.11)	72813(215.84)	85844(235.1)	101651(265.97)	410508(204.01)	<0.001

Inc/10^5; Incidence per 100,000 inhabitants. P value for time trend using Poisson Regression analysis adjusted by age as appropriate. COPD; Chronic Obstructive Pulmonary Disease.

Supplementary Table 3. Time trends in comorbidities, procedures and in-hospital outcomes among patients with a principal diagnosis of urinary tract infection in Spain from 2001 to 2018, according to COPD status and sex.

		2001-2003	2004-2006	2007-2009	2010-2012	2013-2015	2016-2018	p-value
**Men with COPD**	Age, mean(SD)	77.19(8.93)	77.63(8.93)	78.63(8.76)	78.82(8.96)	78.85(9.23)	79.27(9.42)	<0.001
	CCI mean (SD)	0.92(0.83)	1.05(0.88)	1.1(0.86)	1.18(0.89)	1.23(0.89)	1.48(1.03)	<0.001
	CCI =0	1480(38.02)	1652(33.64)	1802(30.65)	1967(27.48)	2052(25.62)	1854(20.33)	<0.001
	CCI 1-2	1531(39.33)	1877(38.22)	2269(38.59)	2791(38.99)	3097(38.67)	3170(34.76)	
	CCI>2	882(22.66)	1382(28.14)	1809(30.77)	2400(33.53)	2860(35.71)	4096(44.91)	
	Urinary catheter, n (%)	196(5.03)	311(6.33)	540(9.18)	634(8.86)	849(10.6)	818(8.97)	<0.001
	LOHS, median (IQR)	7(7)	7(7)	6(6)	6(6)	6(5)	6(5)	<0.001
	IHM, n (%)	252(6.47)	292(5.95)	384(6.53)	383(5.35)	377(4.71)	487(5.34)	<0.001
**Men without COPD**	Age, mean (sd)	70.96(13.3)	71.57(13.21)	72.54(13.12)	73.3(13.03)	73.51(13.06)	74.27(12.94)	<0.001
	CCI mean (sd)	0.8(0.78)	0.9(0.83)	0.98(0.86)	1.07(0.88)	1.09(0.88)	1.17(0.96)	<0.001
	CCI =0	13015(44.36)	13932(40.2)	15841(36.75)	16117(32.48)	18857(31.41)	22317(30.89)	<0.001
	CCI 1-2	10844(36.96)	12874(37.14)	16149(37.47)	18890(38.06)	23106(38.49)	25964(35.94)	
	CCI>2	5483(18.69)	7854(22.66)	11113(25.78)	14621(29.46)	18063(30.09)	23968(33.17)	
	Urinary catheter, n (%)	1445(4.92)	1964(5.67)	3112(7.22)	3947(7.95)	5474(9.12)	5584(7.73)	<0.001
	LOHS, median (IQR)	6(7)	6(7)	6(7)	6(6)	5(6)	5(6)	<0.001
	IHM, n (%)	1443(4.92)	1667(4.81)	2151(4.99)	2252(4.54)	2390(3.98)	3026(4.19)	<0.001
**Women with COPD**	Age, mean (sd)	78.81(9.96)	79.46(10.27)	80.11(10.56)	80.44(10.69)	80.08(11.01)	79.81(11.4)	0.003
	CCI mean (sd)	0.92(0.79)	1.04(0.86)	1.11(0.84)	1.16(0.85)	1.19(0.88)	1.34(0.96)	<0.001
	CCI =0	371(36.81)	359(32.67)	433(28.85)	514(27.31)	534(26.59)	576(23.29)	<0.001
	CCI 1-2	404(40.08)	429(39.04)	605(40.31)	741(39.37)	780(38.84)	905(36.6)	
	CCI>2	233(23.12)	311(28.3)	463(30.85)	627(33.32)	694(34.56)	992(40.11)	
	Urinary catheter, n (%)	21(2.08)	33(3)	52(3.46)	78(4.14)	93(4.63)	100(4.04)	0.009
	LOHS, median (IQR)	7(8)	7(7)	6(7)	6(6)	6(6)	6(6)	<0.001
	IHM, n (%)	57(5.65)	84(7.64)	87(5.8)	120(6.38)	98(4.88)	114(4.61)	0.004
**Women without COPD**	Age, mean (sd)	72.61(14.2)	73.42(14.28)	74.88(14.4)	76(14.23)	76.27(14.47)	77.26(14.47)	<0.001
	CCI mean (sd)	0.81(0.76)	0.88(0.8)	0.95(0.83)	1.02(0.85)	1.05(0.86)	1.14(0.94)	<0.001
	CCI =0	17730(43.3)	19844(40.44)	22435(37.28)	24759(34)	28677(33.41)	32067(31.55)	<0.001
	CCI 1-2	15430(37.69)	18394(37.48)	22793(37.87)	27809(38.19)	32298(37.62)	36794(36.2)	
	CCI>2	7784(19.01)	10833(22.08)	14957(24.85)	20245(27.8)	24869(28.97)	32790(32.26)	
	Urinary catheter, n (%)	734(1.79)	1148(2.34)	1908(3.17)	2739(3.76)	3795(4.42)	3544(3.49)	<0.001
	LOHS, median (IQR)	6(6)	6(6)	6(7)	6(6)	6(6)	6(6)	<0.001
	IHM, n (%)	2014(4.92)	2415(4.92)	3205(5.33)	3550(4.88)	3929(4.58)	5099(5.02)	<0.001

COPD; Chronic Obstructive Pulmonary Disease. CCI; Charlson Comorbidity Index. LOHS; Length Of Hospital Stay. IQR; Inter Quartile Range. IHM; In Hospital Mortality. P-value <0.05 to assess time trend from 2001 to 2018, using bivariate logistic regression (proportions), ANOVA (means), or Kruskal-Wallis test (medians).

Supplementary Table 4. Time trends **in i**solated pathogens codified in hospital admissions among patients with a principal diagnosis of urinary tract infection in Spain from 2001to 2018, according to COPD status and sex.

		2001-2003	2004-2006	2007-2009	2010-2012	2013-2015	2016-2018	p-value
**Men with COPD**	*Enterococcus,* n (%)	116(2.98)	162(3.3)	241(4.1)	352(4.92)	523(6.53)	681(7.47)	<0.001
	Staphylococcus aureus, n (%)	87(2.23)	139(2.83)	167(2.84)	183(2.56)	199(2.48)	246(2.7)	0.399
	*Klebsiella pneumoniae,*n (%)	68(1.75)	129(2.63)	165(2.81)	297(4.15)	544(6.79)	778(8.53)	<0.001
	Escherichia coli, n (%)	738(18.96)	1053(21.44)	1382(23.5)	1888(26.38)	2195(27.41)	2593(28.43)	<0.001
	Proteus, n (%)	65(1.67)	91(1.85)	139(2.36)	178(2.49)	218(2.72)	262(2.87)	<0.001
	Pseudomonas aeruginosa, n (%)	153(3.93)	260(5.29)	336(5.71)	434(6.06)	564(7.04)	608(6.67)	<0.001
**Men without COPD**	*Enterococcus,* n (%)	733(2.5)	1020(2.94)	1498(3.48)	2201(4.43)	3453(5.75)	4271(5.91)	<0.001
	Staphylococcus aureus, n (%)	683(2.33)	884(2.55)	1175(2.73)	1335(2.69)	1595(2.66)	1811(2.51)	0.006
	*Klebsiella pneumoniae,*n (%)	486(1.66)	786(2.27)	1363(3.16)	2209(4.45)	4014(6.69)	6104(8.45)	<0.001
	Escherichia coli, n (%)	5642(19.23)	7393(21.33)	9941(23.06)	13199(26.6)	16919(28.19)	20335(28.15)	<0.001
	Proteus, n (%)	602(2.05)	773(2.23)	1096(2.54)	1346(2.71)	1930(3.22)	2532(3.5)	<0.001
	Pseudomonas aeruginosa, n (%)	1135(3.87)	1526(4.4)	2214(5.14)	3052(6.15)	3929(6.55)	4263(5.9)	<0.001
**Women with COPD**	*Enterococcus,* n (%)	17(1.69)	26(2.37)	41(2.73)	54(2.87)	83(4.13)	116(4.69)	<0.001
	Staphylococcus aureus, n (%)	14(1.39)	18(1.64)	28(1.87)	35(1.86)	29(1.44)	42(1.7)	0.863
	*Klebsiella pneumoniae,*n (%)	13(1.29)	21(1.91)	43(2.86)	93(4.94)	118(5.88)	205(8.29)	<0.001
	Escherichia coli, n (%)	289(28.67)	360(32.76)	536(35.71)	657(34.91)	760(37.85)	1038(41.97)	<0.001
	Proteus, n (%)	28(2.78)	18(1.64)	39(2.6)	46(2.44)	41(2.04)	79(3.19)	0.067
	Pseudomonas aeruginosa, n (%)	18(1.79)	27(2.46)	29(1.93)	41(2.18)	53(2.64)	70(2.83)	0.315
**Women without COPD**	*Enterococcus,* n (%)	570(1.39)	891(1.82)	1436(2.39)	2089(2.87)	2973(3.46)	3960(3.9)	<0.001
	Staphylococcus aureus, n (%)	582(1.42)	773(1.58)	1067(1.77)	1170(1.61)	1378(1.61)	1338(1.32)	<0.001
	*Klebsiella pneumoniae,*n (%)	693(1.69)	1159(2.36)	1860(3.09)	3089(4.24)	5190(6.05)	7647(7.52)	<0.001
	Escherichia coli, n (%)	11039(26.96)	14380(29.3)	18627(30.95)	25249(34.68)	31909(37.17)	38237(37.62)	<0.001
	Proteus, n (%)	848(2.07)	1079(2.2)	1483(2.46)	1988(2.73)	2564(2.99)	3080(3.03)	<0.001
	Pseudomonas aeruginosa, n (%)	614(1.5)	857(1.75)	1217(2.02)	1731(2.38)	2129(2.48)	2274(2.24)	<0.001

COPD; Chronic Obstructive Pulmonary Disease. CCI; Charlson Comorbidity Index. LOHS; Length Of Hospital Stay. IQR; Inter Quartile Range. IHM; In Hospital Mortality. P-value <0.05 to assess time trend from 2001 to 2018, using bivarite logistic regression (proportions), ANOVA (means), or Kruskal-Wallis test (medians) P-value <0.05 to assess time trend from 2001 to 2018, using bivarite logistic regression (proportions).
